# Anti-HIV-1 protease activity of the crude extracts and isolated compounds from *Auricularia polytricha*

**DOI:** 10.1186/s12906-019-2766-3

**Published:** 2019-12-05

**Authors:** Chanin Sillapachaiyaporn, Sunita Nilkhet, Alison T. Ung, Siriporn Chuchawankul

**Affiliations:** 1 Program in Clinical Biochemistry and Molecular Medicine, Department of Clinical Chemistry, Faculty of Allied Health Sciences, Chulalongkorn University, Bangkok, 10330 Thailand; 2 School of Mathematical and Physical Sciences, Faculty of Science, The University of Technology Sydney, Sydney, NSW 2007 Australia; 3 Department of Transfusion Medicine and Clinical Microbiology, Faculty of Allied Health Sciences, Chulalongkorn University, Bangkok, 10330 Thailand; 4 Immunomodulation of Natural Products Research Group, Chulalongkorn University, Bangkok, 10330 Thailand

**Keywords:** *Auricularia polytricha*, HIV-1 protease inhibitor, Human immunodeficiency virus

## Abstract

**Background:**

Acquired immunodeficiency syndrome (AIDS) is caused by the Human immunodeficiency virus type-1 (HIV-1). HIV-1 protease (HIV-1 PR) is an essential enzyme for the HIV replication, and therefore, it is an important target for antiretroviral drugs development, particularly from natural products. *Auricularia polytricha* (AP) is an edible mushroom with several important therapeutic properties. These properties will be investigated as HIV-1 PR inhibitors.

**Methods:**

The sequential hexane (APH), ethanol (APE) and water (APW) extracts from AP were screened for inhibitory activity against HIV-1 PR. The extract that consistently showed the strong HIV-1 PR inhibition was further investigated for its phytochemical constituents. The compounds were purified by column chromatography. The isolated compounds were structurally elucidated using 1D and 2D NMR, HRMS, FTIR, and GC/MS techniques. Each compound was screened against HIV-1 PR to determine its inhibitory activity and to provide an explanation for the activity found in the extract.

**Results:**

Hexane crude extract of AP (APH) exhibited significant inhibition on HIV-1 PR activity. Four major compounds isolated from APH fraction were identified to be two triacylglycerols, linoleic acid and ergosterol. Moreover, all four compounds showed significant inhibition of HIV-1 PR activity.

**Conclusion:**

The findings from this study suggest that AP is a good source of fatty esters, fatty acids and ergosterol. These natural products exhibit anti-HIV-1 properties by blocking HIV-1 PR. These important biological results warrant further development of AP as an alternative antiretroviral drug.

## Background

Human immunodeficiency virus (HIV) is classified into two types: HIV-1 and HIV-2. The HIV-1 is more virulent and transmissible HIV-2 [1, 2]. HIV-1 protease (HIV-1 PR) is an essential enzyme required by the virus for replication. In HIV-1 life cycle, HIV-1 PR plays a role in viral maturation step by cleaving Gag and Gag-Pol polyproteins to structural proteins such as matrix, capsid (p24), nucleocapsid and viral enzymes. At present, many HIV-1 PR inhibitors are available; however, these drugs have adverse side effects [3, 4] and have become ineffective due to drug-resistant [5] and Hence, searching for novel inhibitors from food sources is an innovative approach to combat HIV.

*Auricularia polytricha* (AP) is an edible mushroom that has been reported to have several therapeutic properties such as anti-proliferative [6], anti-oxidant [7, 8] and hypoglycemic activities [9]. The alcoholic and dichloromethane extracts of AP [10, 11] were reported to contain few phenolic compounds and triterpenoids such as cerevistrol. Oleanolic acid, uvaol, ursolic acid, maslinic acid and 2α, 19α-dihydroxy-3-oxo-12-ursen-28-oic acid are triterpenoids. These compounds were also found in other natural sources, and are known to inhibit HIV-1 PR [12–14]. In the case of AP, the anti-HIV-1 activity of its extracts or isolated phytochemicals is unknown. Due to its non-toxicity, phytochemicals and therapeutic properties, further investigation and development of AP as HIV-1 PR inhibitor are warranted. The findings could provide useful insight for antiretroviral drugs development and value-added to this mushroom.

## Methods

### Mushroom species verification

AP fruiting bodies were collected from a Chang Daeng mushroom farm in Prapradaeng, Samutprakarn, Thailand. The identity of the mushroom was confirmed by the DNA sequence similarity of the internal transcribed spacer (ITS) region of ribosomal RNA [15]. DNA was extracted using the method reported by Luangsuphabool et al. [16]. The extracted DNA was submitted to Bioneer sequencing service (Bioneer Corporation, Korea) [17] for polymerase chain reaction (PCR) amplification using primer pair ITS1/ITS4, then analyse the DNA sequence. The mushroom species was identified by comparing nucleotide sequences database on GenBank.

### Crude extracts preparation

The sun-dried *A. polytricha* (AP) was grounded into a powder. Macerations were performed in sequential steps to provide the three crude extracts. AP powder (1 kg) was first macerated twice with hexane (10 L) in an incubator shaker at 225 rpm, at room temperature for 72 h. The combined hexane layers were filtered, and the solvent was evaporated under reduced pressure at 40 °C to give a crude hexane extract (APH) as a thick yellow paste (3.90 g). The dry residue from step 1 was extracted twice with ethanol (10 L) under the same conditions to give the crude ethanol extract (APE) as a dark purple thick paste (4.74 g). Lastly, the dry residue from step 2 was extracted with water (10 L) with stirring at 4 °C for 72 h. The combined water extracts were filtered, and water was removed by freeze-drying to give crude water extract (APW) as a thick brown paste (19.88 g).

### HIV-1 protease inhibitor assay

AP extracts (1 mg/ml) were evaluated using HIV-1 Protease Inhibitor Screening Kit (Fluorometric) (Biovision Incorporated, CA, USA). Pepstatin (1 mM) and DMSO (1%, v/v) were used as a positive control and solvent control, respectively. The assay was performed according to the manufacturer’s instruction. Then the HIV-1 protease fluorescent substrate was added and measured fluorescence (Excitation/Emission = 330/450 nm) in a kinetic-mode for 90 min at 37 °C using PerkinElmer EnSpire plate reader.

### Cell culture

3 T3-L1 cells, normal mouse fibroblasts were maintained in DMEM supplemented with 10% (v/v) BCS at 37 °C in a humidified incubator with 5% of CO_2_.

### Cytotoxicity assay

All extracts and their identified compounds were tested for toxicity on 3 T3-L1 cells by MTT method. The extracts were dissolved in 0.1% (v/v) of dimethyl sulfoxide (DMSO) at varying concentrations (0.03–1.00 mg/mL). The 3 T3-L1 cells (5 × 10^3^ cells, 100 μl) were seeded in each well of 96-well plate overnight to let the cell settle on the plate. Then the cells were treated with the compounds (100 μl) for 24, 48 and 72 h. The DMSO at 0.1% (v/v) and the untreated cell conditions were used as vehicle control and normal control, respectively. At the end of each incubation periods, MTT reagent (20 μl) was added. After 3 hours of incubation, the supernatant was removed then the water-insoluble formazan was dissolved in DMSO (150 μl). The absorbance of the converted dye was measured at a wavelength of 570 nm. The results were reported in CC_50_ values, calculated by standard curve analysis of four parameters logistic in Sigma plot 12 software.

### Statistical analysis

All experiments were performed in triplicate*s* for each condition. The results were presented as the mean with the standard error of the mean (mean ± SEM) of three independent experiments. Statistic significant was analysed using one-way ANOVA following Dunnett’s test by SPSS 16.0 software. The *P* values less than 0.05 were considered statistically significant.

### Purification of APH fraction

APH crude extract was analysed using TLC silica plate (silica gel 60 F254, Merck), using hexane/ethyl acetate (80:20, v/v, analytical grade) as mobile phases to reveal four separate spots, referring as fraction 1 (F1), fraction 2 (F2), fraction 3 (F3) and fraction (F4).

The APH crude extract (650.2 mg) was dissolved in ethyl acetate to induce the crystallisation of F4 from the mixture. The crystals (15.7 mg) were filtered and dried. The mother liquor was collected, and the solvent was removed to give the thick oil (634.5 mg) which was further purified using flash silica gel 60 column chromatography (hexane/ethyl acetate, 80:20, v/v) to give three fractions, F1 + F2 (392.0 mg), F3 (118.0 mg) and F4 (116.0 mg).

### Preparative liquid chromatography (prep LC)

The pre-crystallisation APH crude mixture (450.0 mg) was purified by Reveleris® prep purification system with Reveleris® silica flash cartridge (24 g) using hexane/ethyl acetate (80:20, v/v) as a mobile phase at flow rate of 32 ml/min to give F1 + F2 (168.2 mg), F1 + F2 + F3 (142.3 mg), F3 (52.7 mg) and F4 (75.4 mg). The mixture of F1 + F2 (168.2 mg) was further purified by Reveleris® prep system using hexane/ethyl acetate (95:5, v/v) as a mobile phase to give F1 (149.9 mg) and F2 (2.2 mg). The mixture of F1 + F2 + F3 (20 mg) was purified by TLC plate silica gel 60 F254, using hexane/ethyl acetate (80:20, v/v) as a mobile phase. The band of F2 (1.2 mg) was purely isolated.

### Compound identification

Isolated compounds were structurally elucidated using NMR, FTIR, HRMS and GC/MS techniques. The ^1^H (500 MHz), ^13^C (125 MHz) and 2D NMR: COSY and DEPT NMR spectra were recorded on a 500 MHz Agilent spectrometer in deuterated chloroform (CDCl_3_). The FTIR analyses were performed using Nicolet 6700 FTIR spectrometer (Thermo scientific). High-resolution mass spectra were obtained using an Agilent 6510 Q-TOF Mass Spectrometer (ESI). The GC/MS analyses were performed using Agilent 6870/5973n MS (EI) spectrometer. The peaks were identified using the mass spectral library available in the software.

#### Fraction 1 (F1)

*R*_*f*_ value (TLC): 0.88 (ethyl acetate/hexane, 20:80). HRMS (ESI): m/z 884.5865 [M]^+^ (calculated for C_57_H_104_O_6_ = 884.7833). FTIR (cm ^− 1^): 2921.41, 2852.12 (C-H) and 1742.29 (C=O). ^1^H NMR δ_H_ (ppm): 5.349 (m, C*H*=C*H*), 5.264 (m, C*H*), 4.286 (dd, C*H*_*2*_), 4.151 (dd, C*H*_*2*_), 2.770 (t, =CH-C*H*_*2*_-CH=), 2.315 (t, C*H*_*2*_CO_2_), 2.042 (m, C*H*_*2*_CH=CH), 1.607 (m, C*H*_*2*_CH_2_CO_2_), 1.254 (m, C*H*_*2*_) and 0.881 (t, C*H*_*3*_). ^13^C NMR (CDCl_3_, 125 MHz) δ_C_ (ppm): 173.306, 173.261, 172.859 (C), 130.240, 130.039, 130.023, 129.697, 128.077, 127.910 (CH), 68.891 (CH), 62.106, 62.104 (CH_2_), 34.212, 34.068, 34.038, 31.940, 31.917, 31.537, 29.781, 29.720, 29.716, 29.675, 29.641, 29.625, 29.622, 29.542, 29.496, 29.375, 29.360, 29.345, 29.330, 29.288, 29.212, 29.193, 29.147, 29.136, 29.098, 29.064, 27.239, 27.213, 27.211, 27.190, 25.642, 24.898, 24.879, 24.849, 22.702, 22.694, 22.584 (CH_2_), 14.128, 14.082 (CH_3_).

#### Fraction 2 (F2)

R_f_ value (TLC): 0.61 (ethyl acetate/hexane, 20:80). HRMS (ESI): m/z 862.6072 [M]^+^ (calculated for C_55_H_106_O_6_ = 862.7989). FTIR (cm ^− 1^): 2960.97, 2913.33, 2849.18 (C-H) and 1735.55 (C=O). ^1^H NMR δ_H_ (ppm): 5.349 (m, C*H*=C*H*), 5.264 (m, C*H*), 4.282 (dd, C*H*_*2*_), 4.148 (dd, C*H*_*2*_), 2.769 (t, =CH-C*H*_*2*_-CH=), 2.323 (t, C*H*_*2*_CO_2_), 2.040 (m, C*H*_*2*_CH=CH), 1.598 (m, C*H*_*2*_CH_2_CO_2_), 1.253 (m, C*H*_*2*_) and 0.880 (t, C*H*_*3*_).

#### Fraction 3 (F3)

R_f_ value (TLC): 0.44 (ethyl acetate/hexane, 20:80). HRMS (ESI): m/z 281.2468 [M + H]^+^ (calculated for C_18_H_33_O_2_ = 281.2481). FTIR (cm ^− 1^): 2955.28, 2914.71, 2847.62 (C-H), 1699.69 (C=O), 1471.38, 1462.96 and 1429.69 (C=C). ^1^H NMR δ_H_ (ppm) 5.344 (m, C*H*=C*H*), 2.771 (t, =CH-C*H*_*2*_-CH=), 2.345 (t, C*H*_*2*_CO_2_), 2.042 (m, C*H*_*2*_CH=CH), 1.631 (m, C*H*_*2*_CH_2_CO_2_), 1.255 (m, C*H*_*2*_) and 0.880 (t, C*H*_*3*_).

#### Fraction 4 (F4)

R_f_ value (TLC): 0.27 (ethyl acetate/hexane, 20:80). HRMS (ESI): m/z 395.3303 [M-H]^+^ (calculated for C_28_H_43_O = 395.3314). GCMS (EI) m/z 396 [M]^+^. FTIR spectra (cm^− 1^) 3414.02 (O-H); 2952.17, 2928.38 and 2868.82 (C-H) and 1655.20 (C=C). ^1^H NMR spectra δ_H_ (ppm): 5.575 (dd, 1H), 5.385 (m, 1H), 5.205 (m, 1H), 3.639 (m, 2H), 2.459 (ddd, 2H), 2.284 (t, 2H); the position of this signal varied from 1.250–2.080 ppm in the other saturated methylene and methine protons (total 18H); 1.044 (d, 3H), 0.948 (s, 3H), 0.925 (d, 3H), 0.833 (t, 6H) and 0.632 (s, 3H). ^13^C NMR δ_C_ (ppm): 141.351, 139.769 (C), 135.551, 131.962, 119.573, 116.273, 70.457, 55.728, 54.555, 46.244 (CH), 42.830 (C), 42.815 (CH), 40.797 (CH_2_), 40.418 (CH), 39.082, 38.373 (CH_2_), 37.026 (C), 33.085 (CH), 31.997, 28.283, 22.991, 21.110 (CH_2_), 21.098, 19.949, 19.642, 17.601, 16.281 and 12.047 (CH_3_).

### Base hydrolysis of fraction 1 (F1)

Methanolic sodium hydroxide solution (0.5 M, 5 mL) was added to F1 (110 mg). The mixture was heated with stirring at 90 °C for 10 min. The reaction was cooled in an ice bath, then methanol (5 mL) was added. The resulting mixture was heated at 90 °C for 10 min. Upon cooling, the pH of the reaction mixture was adjusted to zero by adding 1 M of HCl (10 mL). The acidic solution was extracted with dichloromethane (3 × 15 mL). The combined extracts were dried over anhydrous sodium sulfate and filtered. The dichloromethane was removed under reduced pressure to give a crude product (107 mg), which was analysed by GC/MS.

## Results

### A.polytricha (AP) extraction

*A.polytricha* (AP) was sequentially extracted; first with hexane, then with ethanol and finally with cold water. Percentage yield by weight of each extract from the dry weight of AP is tabulated in Table 1.

**Table 1:** Percentage of crude extracts

Crude extract	%Yield
APH	0.39
APE	0.47
APW	1.99

### HIV-1 PR inhibitory activity of crude extracts

**Fig. 1:**
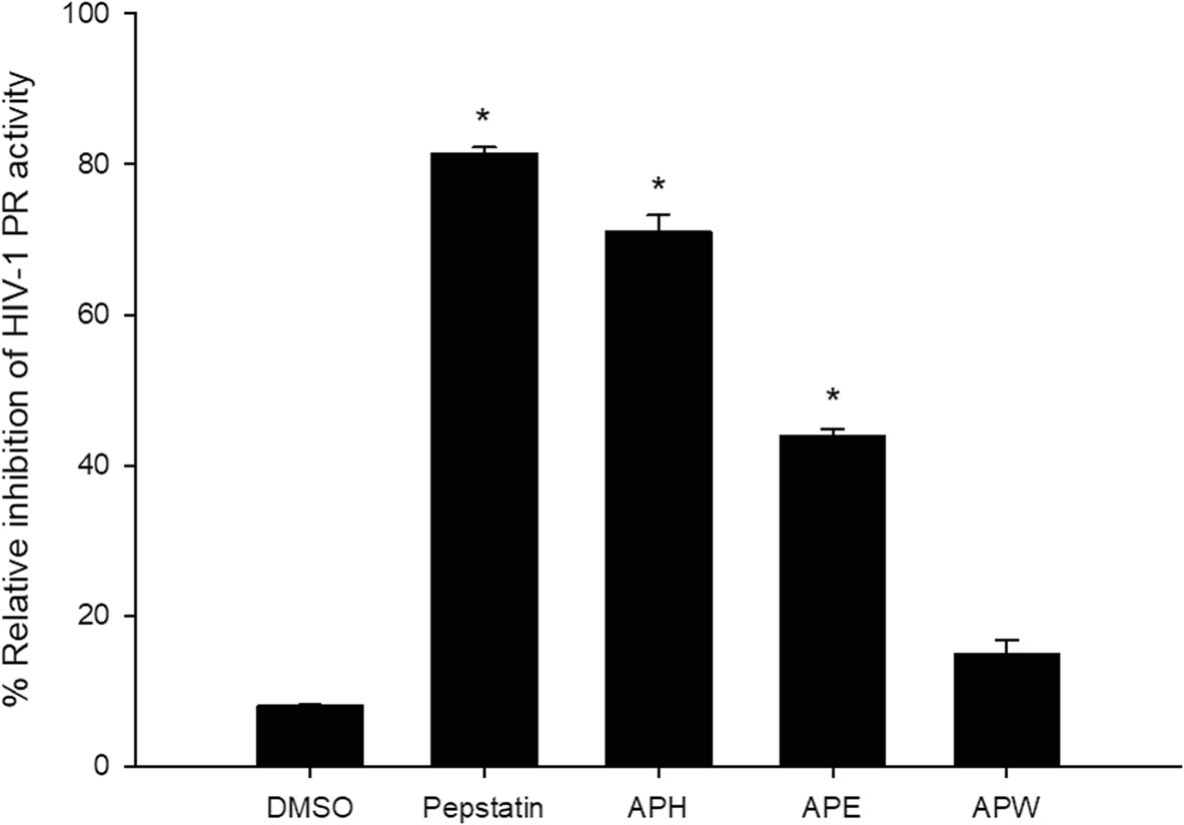
Percentage of inhibitory on HIV-1 PR activity of DMSO (1.0%, v/v), pepstatin (1 mM) and crude AP extracts (1 mg/ml). The error bars indicate the standard error of the means. *p < 0.05 compared to vehicle control

Crude extracts were screened at 1 mg/mL against HIV-1 PR. DMSO (1%, v/v) and pepstatin (1 mM) were used as controls, which showed the percentage of inhibition at 8.07 ± 0.13 and 81.48 ± 0.76, respectively. APH exhibited the highest percentage of inhibitory activity of 71.07 ± 2.17, followed by APE (43.82 ± 1.04%) and APW (14.80 ± 1.96%) compared to vehicle control. (Fig. 1 and Table 2).

**Table 2:** Percent relative inhibition and half-maximal inhibition concentration (IC_50_) on HIV-1 PR activity of AP crude extracts, pepstatin and DMSO

Sample	%Relative inhibition	IC_50_
APH	71.07 ± 2.17^a^	0.80 ± 0.08 mg/ml
APE	43.82 ± 1.04^a^	n/a
APW	14.80 ± 1.96^a^	n/a
Pepstatin	81.48 ± 0.76^b^	0.32 ± 0.05 mM
DMSO	8.07 ± 0.13^c^	n/a

Samples were tested at ^a^1 mg/ml, ^b^1 mM and ^c^1% (v/v). *n/a* Not applicable

APH and APE show significant inhibition more strongly in HIV-1 PR assay than that of APW. In addition, APH has the most potent activity against HIV-PR with 50% inhibitory concentration (IC_50_) of 0.80 ± 0.08 mg/ml (Table 2).

### Cytotoxicity of APH crude extract

**Fig. 2:**
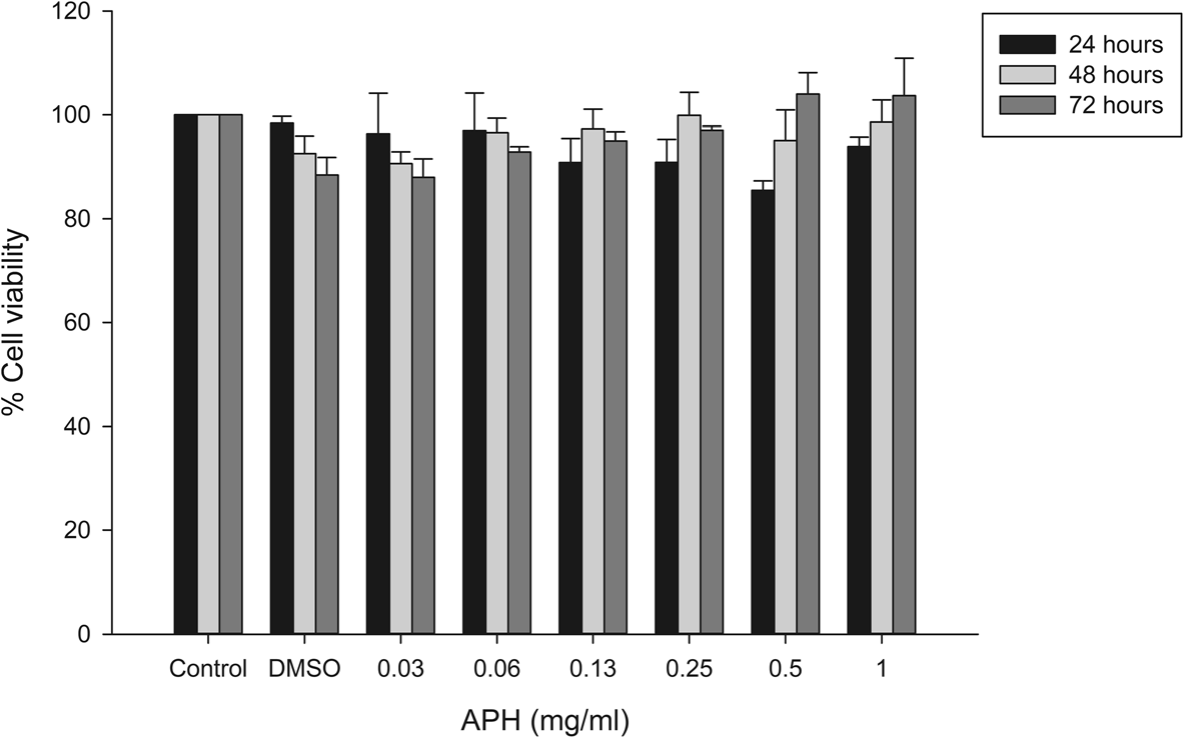
Cytotoxicity of APH against 3 T3-L1 cells at 24, 48 and 72 h. The error bars indicate the standard error of the means. *p < 0.05 compared to vehicle control

In vitro maximum safe concentration of APH crude extract against normal mammalian fibroblasts, the 3 T3-L1 cells were determined by MTT assay for 24 to 72 h. APH showed no significant cytotoxicity on the fibroblasts when treated with a high concentration of APH, 1.00 mg/mL. In addition, all treated concentrations exhibited percentage cell viability higher than 85% (Fig. 2). In comparison, APH showed the HIV-1 PR inhibitory activity (IC_50_ = 0.80 ± 0.08 mg/ml), at the concentration slightly less than cytotoxic concentration (> 1.00 mg/ml). These results suggest that APH could be safely used for in vitro HIV-1 PR inhibition.

### Isolation and purification of APH

TLC analysis of APH (80:20, v/v hexane: ethyl acetate) revealed four fractions F1, F2, F3 and F4 with the retention factor (R_f_) of 0.88, 0.61, 0.44 and 0.27, respectively (see Additional file 1: Figure S1). The F1, F2 and F4 were visible under UV light (254 nm) while the F3 was detectable at a long wavelength (365 nm).

**Fig. 3:**
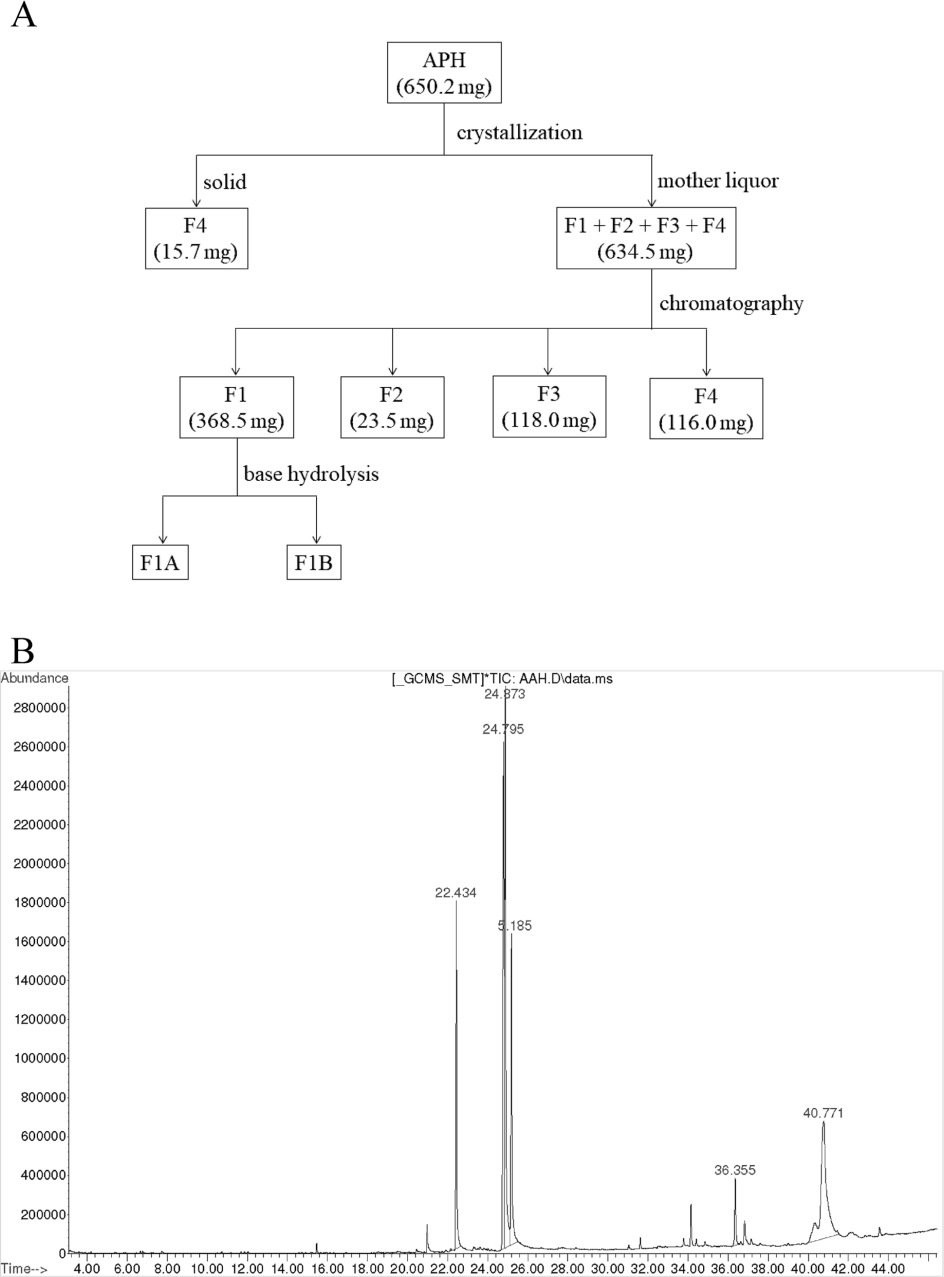
APH isolation and identification. **a** Schematic representation of APH isolation. **b** Gas chromatogram of APH

The crude APH (650.2 mg) was dissolved in ethyl acetate to recrystallise out pure F4 (15.7 mg) as white needles form. The crude mixture recovered from the mother liquor was further purified using the Reveleris® prep purification system to give F1 (368.5 mg), F2 (25.5 mg), F3 (118 mg) and the additional F4 (116.0 mg) as summarised in Fig. 3a.

### Identification of isolated compounds from APH by GC-MS analysis

The chemical profile of APH was first analysed using GC-MS, which showed five major compounds at a retention time of 22.434, 24.795, 24.873, 25.185 and 40.771 min (Fig. 3b). The corresponding mass spectrometry (EI-MS) analysis [M^+^] found m/z of 256.3, 280.3, 282.3, 284.3 and 396.4. The mass spectral library identified these m/z to be palmitic acid (12.02%), linoleic acid (23.75%), oleic acid (23.23%), stearic acid (12.20%) and ergosterol (25.75%), respectively. Moreover, a group of small peaks was found at retention time varies from 36.00 to 37.00 min with m/z of 376.3, indicating the presence of anthraergostapentene (3.05%).

### Quantitative analysis of chemical constituents in APH

The quantitative analysis of chemical constituents was determined by extrapolating on the standard curve of commercial ergosterol (see Additional file 1: Figure S2). The results showed that at 10 mg/ml of APH was composed of palmitic acid, linoleic acid, oleic acid, stearic acid and ergosterol at concentrations of 0.733, 0.364, 0.679, 0.665 and 0.368 mg/ml, respectively (Table 3). From the quantitative profile analysis, 10 mg of APH crude extract yielded only 2.931 mg of total interested compounds or 29.31% of starting material. This value indicated low productivity of GC-MS analysis; some compounds were lost during the analysis.

**Table 3:** Quantitative analysis of APH analysis by GC-MS

Compound	Quantity (mg/10 mg of APH)	Ratio
Palmitic acid	0.364	1
Linoleic acid	0.679	2
Oleic acid	0.665	2
Steric acid	0.368	1
Anthraergostapentene	0.122	n/a
Ergosterol	0.733	2

*n/a* Not applicable

However, these results correlate with the calculation of the percentage of compounds. There showed a ratio of palmitic acid: linoleic acid: oleic acid: stearic acid: ergosterol is 1: 2: 2: 1: 2 in both percentage and quantitative values. It is noteworthy that the APH crude extract contained anthraergostapentene, which was derived from the ergosterol. This was confirmed by the GC/MS analysis of commercial ergosterol, which also showed the presence of anthraergostapentene with a retention time between 36.00 and 37.00 min (m/z of 376.4).

### Structure elucidation of APH isolated compounds

***F1*** was obtained as a pale-yellow wax. The structure of F1 could be identified as triacylglycerol or fatty ester by its ^1^H NMR signal pattern (see Additional file 1: Figure S3). The FTIR spectrum showed absorption bands at (cm^− 1^) 2921.41 and 2852.12 (C-H) and 1742.29 (C=O). According to Chira et al. report [18], they suggested calculation methods for determining vegetable oils composition using ^1^H NMR spectral data. The chemometric equations were used to determine of oils composition on four classes of fatty acids. These are linolenic acid, linoleic acid, monounsaturated fatty acids (oleic acid) and saturated fatty acids (palmitic acid, stearic acid). The chemometric calculation showed that triacylglycerol of F1 comprised linoleic acid, monounsaturated fatty acids and saturated fatty acids in the same proportion, which could exist in two possible forms, as indicated in Table 4.

**Table 4:** Name, structure and molecular formula of constituent compounds of APH crude extract

Compound	Name	Structure	Molecular formula
F1A	Linoleoyl, oleoyl, palmitoylglycerol		C_55_H_100_O_6_
F1B	Linoleoyl, oleoyl, stearoylglycerol		C_57_H_104_O_6_
F2	Distearoyl, palmitoylglycerol		C_55_H_106_O_6_
F3	Linoleic acid		C_18_H_32_O_2_
F4	Ergosterol		C_28_H_44_O

To confirm the origin and composition of fatty acids, the F1 was hydrolysed under basic conditions. Then hydrolysed crude product was subjected to GC-MS analysis. Gas chromatogram showed four major peaks which are due to including palmitic acid methyl ester (11.19%), linoleic acid methyl ester (25.27%), oleic acid methyl ester (20.99%) and stearic acid methyl ester (12.20%) at the retention time of 21.944, 24.259, 24.338 and 24.677, respectively. Moreover, the palmitic acid (3.56%) and the incomplete hydrolysed product were found in this analysis (see Additional file 2: Table S1). These data indicated that the F1 contains two possible forms of triacylglycerols; form A and B. Form A of triacylglycerols should comprise one palmitic acid, linoleic acid and oleic acid while form B of triacylglycerol comprises a stearic acid, linoleic acid and oleic acid. Two possible combinations were derived from the observed proportion of four fatty acids: palmitic acid, linoleic acid, oleic acid and stearic acid in the GC-MS analysis of hydrolysed F1. The ratio of the four acids was found to be 1: 2: 2: 1. Due to oleic acid was classified as a monounsaturated fatty acid, palmitic acid and stearic acid were classified as saturated fatty acids, the ratio of linoleic acid: monounsaturated fatty acid: saturated fatty acids is 1: 1: 1. These results correlated with the results from the chemometric calculation based on ^1^H NMR analysis, which was mentioned above. The two possible forms also are supported by the abundance of these four acids in the quantitative analysis of APH, which found the ratio between palmitic acid, linoleic acid, oleic acid and stearic acid to be 1: 2: 2: 1.

Furthermore, the hydrolysed F1 was analysed by ^1^H NMR spectroscopy; the ^1^H NMR spectrum (see Additional file 1: Figure S4) showed a signal of protons responding to methyl ester at a chemical shift of 3.666 ppm. These confirmed that fatty acid methyl esters were produced from the hydrolysis reaction and it related to the results of the GC-MS analysis.

***F2*** was obtained as a yellow oil. The structure of F2 could be identified to triacylglycerol by its ^1^H NMR signal pattern, and protons are responding to glycerol backbone at δ_H_ (ppm) 5.264 (1H at β-carbon), 4.282 (2H at α-carbon) and 4.148 (2H at α-carbon). Mass spectrum analysis indicated that the F2 has the molecular formula of C_55_H_106_O_6_; it was confirmed by HRMS (ESI) [M]^+^ m/z 862.6072 (calculated for 862.7989). According to the molecular formula, the F2, triacylglycerol could compose of two stearic acids and one palmitic acid unit in the molecule (Table 4).

***F3*** was obtained as a pale-yellow oil. The structure of F3 could be identified as an unsaturated fatty acid by its ^1^H NMR signal pattern. The ^1^H NMR showed peaks responding to alkene and allylic protons at δ_H_ (ppm) 5.344 (CH=CH) and 2.771 (=CH-CH2-CH=), respectively. The FTIR spectrum showed absorption bands at (cm − 1) 2955.28, 2914.71 and 2847.62 (C-H); 1699.69 (C=O) and 1471.38, 1462.96 and 1429.69 (C=C). The mass spectroscopy data indicated that the F3 has the molecular formula of C_18_H_32_O_2_, it is supported by HRMS (ESI) [M + H]^+^ m/z 281.2468 (calculated for 281.2481). All spectroscopy data indicated that the F3 was identified as linoleic acid (Table 4). F3, therefore, is one of the hydrolysed products from F1.

***F4*** was obtained as a white powder. Spectroscopy data indicated that the F4 has the molecular formula of C_28_H_44_O; it is supported by HRMS (ESI) [M-H]^+^ found m/z 395.3303 (calculated for 395.3314). Moreover, the ^1^H and ^13^C NMR spectra of F4 are in agreement with that reported in the literature for ergosterol [19] (Table 4).

### HIV-1 PR inhibitory activity of APH identified compounds

**Fig. 4:**
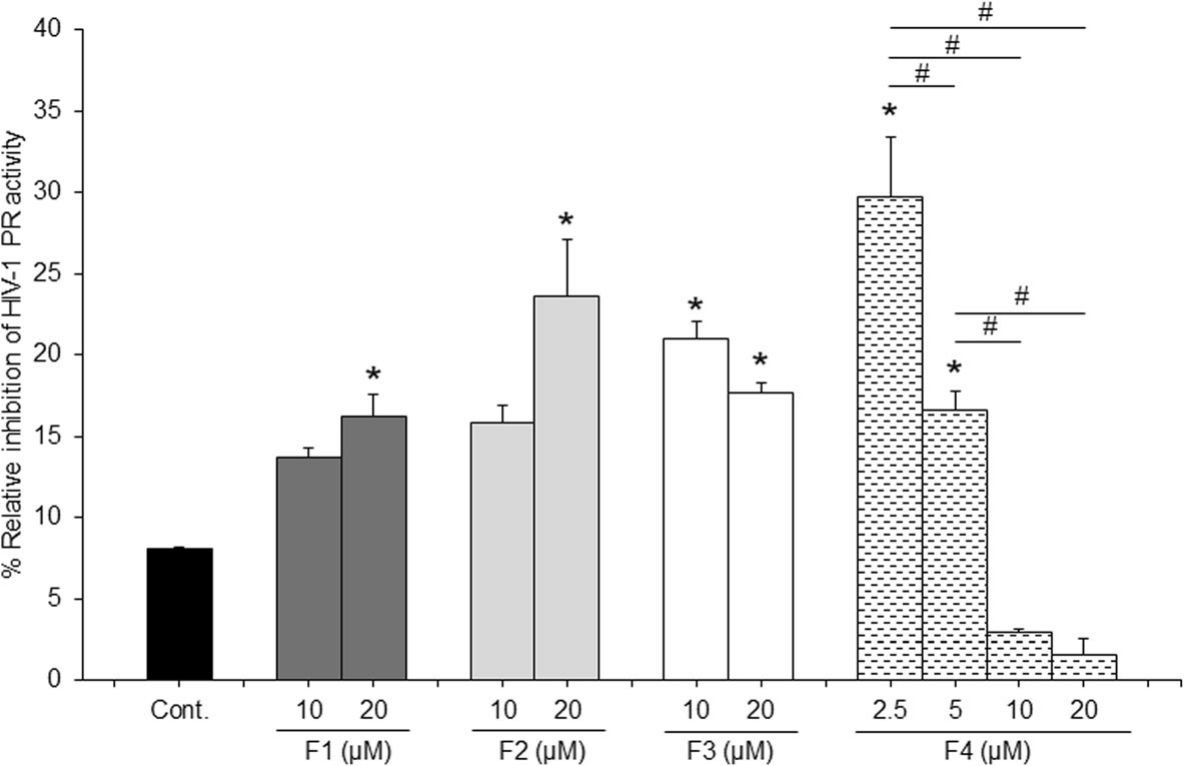
Percent relative inhibition of HIV-1 PR activity of identified compounds: F1 (combination of linoleoyl, oleoyl, palmitoylglycerol and linoleoyl, oleoyl, stearoylglycerol), F2 (distearoyl, palmitoylglycerol), F3 (linoleic acid) and F4 (ergosterol). The error bars indicate the standard error of the means. *p < 0.05 compare to vehicle control. # *p* < 0.05 compared to other concentration of the same treatment

Our results have shown that all identified compounds significantly inhibited HIV-1 PR activity. At 20 μM of triacylglycerols (F1 and F2) and linoleic acid (F3) inhibited HIV-1 PR activity with the percentage of relative inhibition range from 13 to 23%. Ergosterol (F4) was inactive against the enzyme at a concentration of ≥5 μM; however, it became significant active at a concentration ≤ 10 μM (Fig. 4).

## Discussion

In this study, we have demonstrated that hexane extract from AP could inhibit HIV-1 replication by blocking HIV-1 PR activity. This further supports by the work of El-Mekkawy et al., which reported that 3β-5α-dihydroxy-6β -methoxyergosta-7,22-diene, found in hexane fraction of methanol extract from *Ganoderma lucidum* could inhibit HIV-1 PR activity [20]. Moreover, hexane extract of *Lignosus rhinocerus* showed strong inhibitory effect on HIV-1 PR [21]. Since AP is a common edible mushroom, compounds presented within the crude extract, therefore are considered to be non-toxic. The phytochemicals that are responsible for the observed inhibitory activity would be valuable compounds for HIV-1 drug discovery. Although there have been a few chemical constituents reported to be isolated from AP. However, to our knowledge, a thorough investigation of phytochemicals with anti-HIV properties from AP is not known. Hence, the investigation of the APH crude extracts has been the main focus of this research.

Four compounds were isolated and purified from APH. Structure elucidation of these four compounds using NMR, IR and HRMS techniques confirmed them to be two triacylglycerols (F1 and F2), linoleic acid (F3) and ergosterol (F4). However, the results from GC-MS revealed five compounds; palmitic acid, linoleic acid (F3), oleic acid, stearic acid and ergosterol (F4). The GC-MS results accounted for F3 and F4 and not for F1 and F2. One can conclude that the presence of unaccounted fatty acids resulted from the high temperature of GCMS that induced hydrolysis of F1 and F2 to form their corresponding fatty acids.

Our finding of three triacylglycerols, linoleic acid and ergosterol in APH is comparable to the previous studies, which showed fungi could be a source of fatty acids and sterol such as palmitic, stearic, oleic and linoleic acid as well as ergosterol [22]. Ruess et al. also reported that many edible mushroom species contain a high proportion of unsaturated fatty acid, particularly linoleic acid [23] that is also a precursor of 1-octen-3-ol; the aromatic compound might give mushroom flavour [24].

Moreover, all identified compounds exhibited HIV-1 PR inhibition, but their activities were lower than the original APH, suggesting that HIV-1 PR inhibitory activity of APH might result from a combined effect of these identified compounds. Therefore, the synergistic effect of the isolated compounds should be evaluated further as a potential HIV-1 PR inhibitor. Further, ergosterol appeared to have a limited concentration range of activity (2.5 to 10 μM). This finding might be due to the solubility limit of ergosterol, in water. The solubility of ergosterol, perhaps improved when it was in the mixture of other components of APH. These results suggest that all identified compounds in APH, worked synergistically to inhibit HIV-1 PR.

Previous reports found that several triterpenoids such as oleanolic acid, uvaol, ursolic acid, maslinic acid and 2α, and 19α-dihydroxy-3-oxo-12-ursen-28-oic acid exhibited anti-HIV-1 PR activity [12–14]. Lee et al. demonstrated that palmitic acid, saturated fatty acid could inhibit viral entry by directly blocking gp120-CD4 complex formation [25]. In addition, linoleic acid and oleic acid could inhibit HIV-1 RT activity in vitro determination [26]. Interestingly, linoleic acid might be a dual HIV-1 enzymes inhibitor that could inhibit not only HIV-1 PR but also RT.

## Conclusions

In conclusion, the screening assays showed that APH and APE inhibited both HIV-1 PR activity. APH provided the most potent inhibitory activity on HIV-1 PR. The chemical analysis revealed that APH was composed of four significant compounds including two triacylglycerols, linoleic acid and ergosterol. For triacylglycerols, they comprise of four corresponding fatty acids: palmitic acid, linoleic acid, oleic acid and stearic acid. Quantitative of fatty acids composition showed a higher proportion of unsaturated fatty acids than saturated fatty acids. All identified compounds significantly inhibited HIV-1 PR activity. These results suggested that AP could be a good source of therapeutic fatty acids and ergosterol, which have the anti-HIV-1 replication. Therefore, our results have provided useful data for an alternative approach to HIV-1 PR drugs development.

## Supplementary information

**Additional file 1: Figure S1.** TLC analysis of APH. **Figure S2.** Standard curve of commercial ergosterol by GC-MS analysis. **Figure S3.** ^1^H NMR spectrum of F1 showing the elucidated core structure and protons responsible for the chemical shifts observed. **Figure S4.** ^1^H NMR spectrum of hydrolysed products from F1, showing a signal of protons responding to methyl ester at the chemical shift of 3.666 ppm.

**Additional file 2: Table S1** List of hydrolysed products and their corresponding fatty acids of F1 hydrolysis from GC-MS analysis.

## Data Availability

All data analysed in this study are included in this manuscript and its supplementary information files.

## Abbreviations

AP: *Auricularia polytricha*
APH: *AP* crude hexane extract
HIV-1 PR: HIV-1 protease
HIV-1: Human immunodeficiency virus type 1
