# Supplementary material for: Anti-HIV-1 protease activity of the crude extracts and isolated compounds from Auricularia polytricha
Source: BMC Complement Altern Med. 2019 Dec 5;19:351. doi: 10.1186/s12906-019-2766-3 (PMC6896332; doi:10.1186/s12906-019-2766-3)
Supplement: Supplementary file 1 — Additional file 1: Figure S1. TLC analysis of APH. Figure S2. Standard curve of commercial ergosterol by GC-MS analysis. Figure S3. 1H NMR spectrum of F1 showing the elucidated core structure and protons responsible for the chemical shifts observed. Figure S4. 1H NMR spectrum of hydrolysed products from F1, showing a signal of protons responding to methyl ester at the chemical shift of 3.666 ppm. [file 12906_2019_2766_MOESM1_ESM.docx]

**Additional file 1**


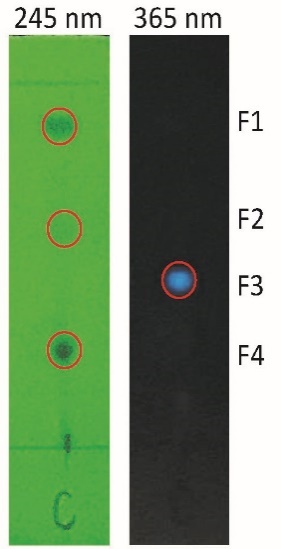


**Figure A1** TLC analysis of APH

**Figure A2** Standard curve of commercial ergosterol by GC-MS analysis


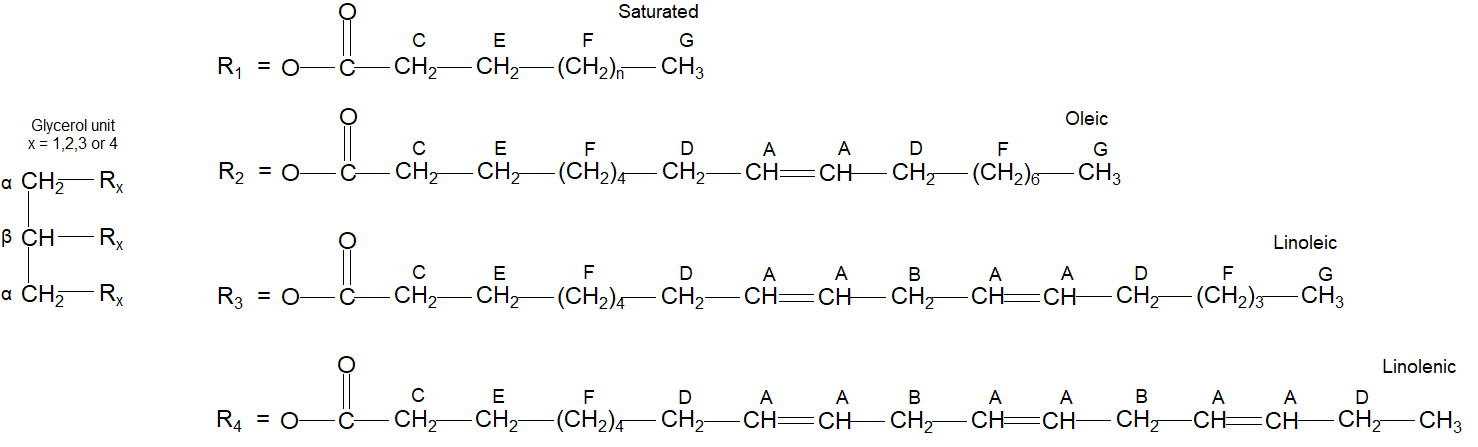

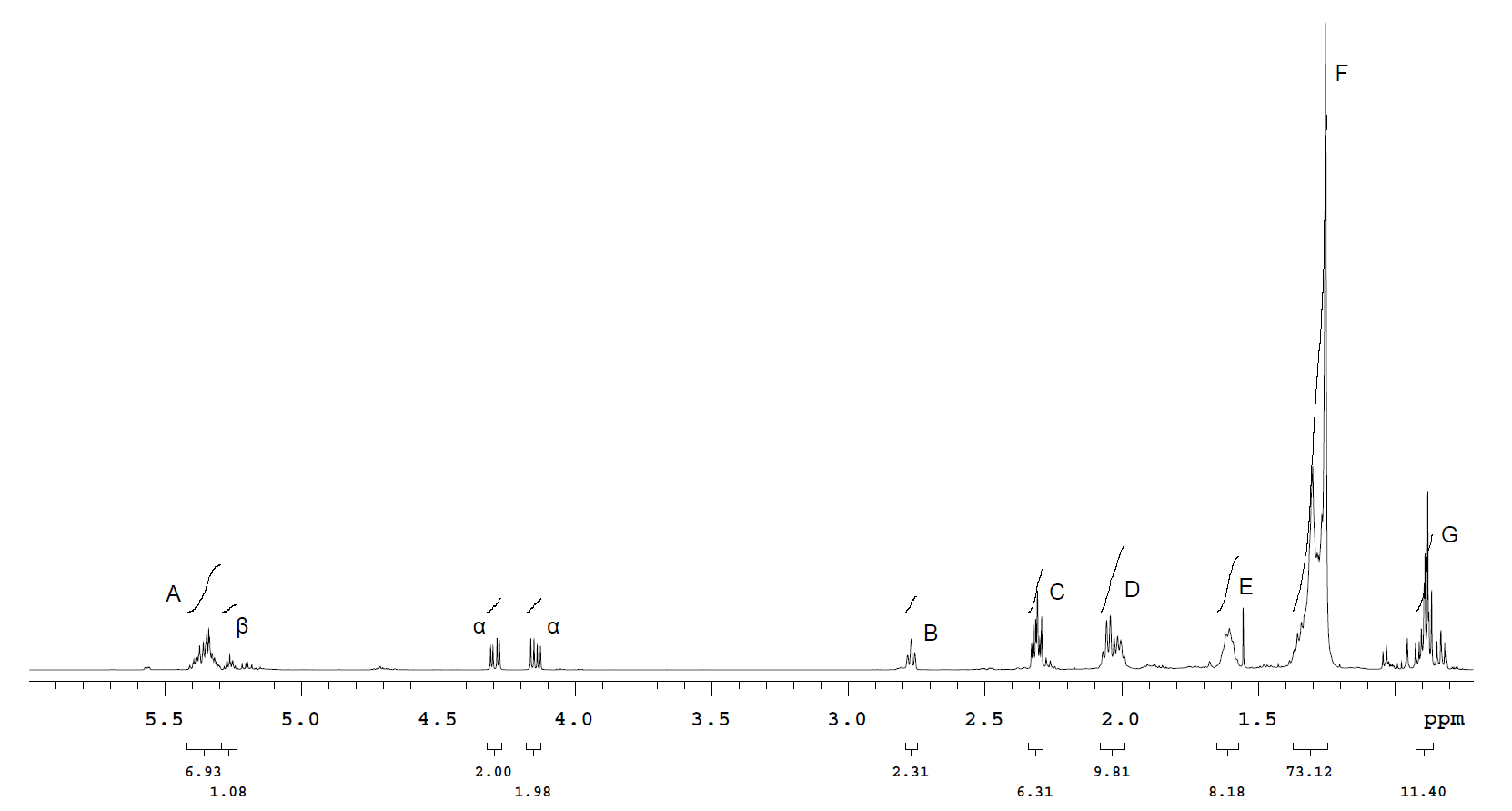


**Figure A3** ^1^H NMR spectrum of F1 showing the elucidated core structure and protons responsible for the chemical shifts observed


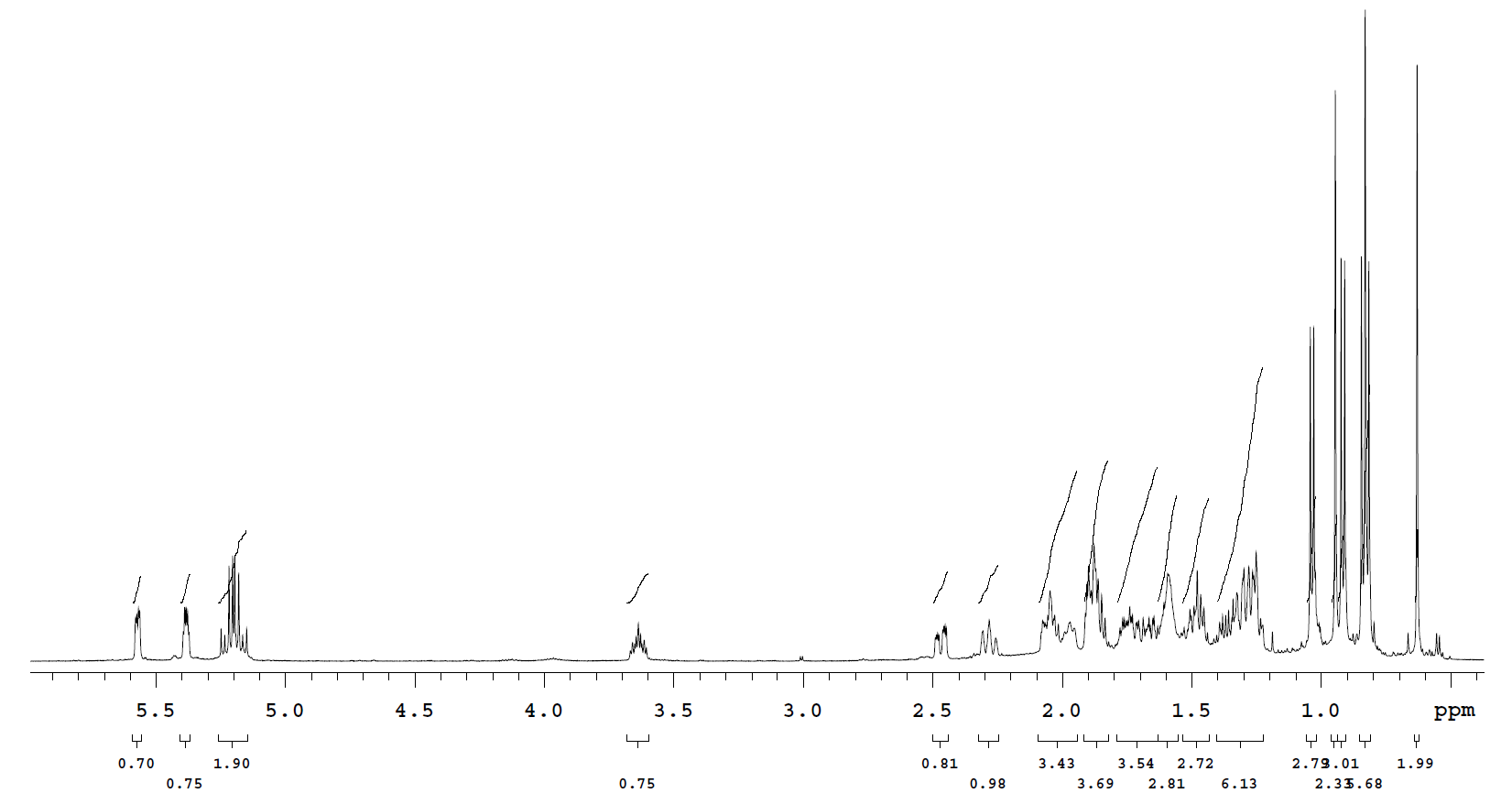


**Figure A4** ^1^H NMR spectrum of hydrolysed products from F1, showing a signal of protons responding to methyl ester at the chemical shift of 3.666 ppm
