# Supplementary material for: Anti-HIV-1 protease activity of the crude extracts and isolated compounds from Auricularia polytricha
Source: BMC Complement Altern Med. 2019 Dec 5;19:351. doi: 10.1186/s12906-019-2766-3 (PMC6896332; doi:10.1186/s12906-019-2766-3)
Supplement: Supplementary file 2 — Additional file 2: Table S1 List of hydrolysed products and their corresponding fatty acids of F1 hydrolysis from GC-MS analysis. [file 12906_2019_2766_MOESM2_ESM.docx]

**Additional file 2**

**Table A1** List of hydrolysed products and their corresponding fatty acids of F1 hydrolysis from GC-MS analysis

| **RT (min)** | **Hit name** | **m/z** | **% Area of total** |
| --- | --- | --- | --- |
| **21.944** | Palmitic acid, methyl ester | 270.3 | 11.188 |
| **22.435** | Palmitic acid | 256.3 | 3.561 |
| **24.259** | Linoleic acid, methyl ester | 294.3 | 25.269 |
| **24.338** | Oleic acid, methyl ester | 296.3 | 20.994 |
| **24.677** | Stearic acid, methyl ester | 298.3 | 12.199 |
